# MicroRNAs as Mediators of Adipose Thermogenesis and Potential Therapeutic Targets for Obesity

**DOI:** 10.3390/biology11111657

**Published:** 2022-11-13

**Authors:** Lunkun Ma, Ankit Gilani, Qian Yi, Liling Tang

**Affiliations:** 1 Key Laboratory of Biorheological Science and Technology, Ministry of Education, College of Bioengineering, Chongqing University, Chongqing 400044, China; 2 Weill Center for Metabolic Health, Cardiovascular Research Institute, Division of Cardiology, Department of Medicine, Weill Cornell Medicine, New York, NY 10021, USA; 3 Department of Physiology, School of Basic Medical Science, Southwest Medical University, Luzhou 646099, China

**Keywords:** microRNAs, obesity, browning, adipose

## Abstract

**Simple Summary:**

Thermogenesis in beige and brown adipose tissue has a significant role in combating metabolic disorders, such as type 2 diabetes and diet-induced obesity. A large number of studies in recent years have demonstrated that microRNAs play an essential role in regulating adipose thermogenesis and offer considerable potential as a critical new target for obesity treatment. In this review, we highlight the diverse roles of microRNAs in adipose thermogenesis and identify their regulatory roles in the development of obesity.

**Abstract:**

Obesity is a growing health problem worldwide, associated with an increased risk of multiple chronic diseases. The thermogenic activity of brown adipose tissue (BAT) correlates with leanness in adults. Understanding the mechanisms behind BAT activation and the process of white fat “browning” has important implications for developing new treatments to combat obesity. MicroRNAs (miRNAs) are small transcriptional regulators that control gene expression in various tissues, including adipose tissue. Recent studies show that miRNAs are involved in adipogenesis and adipose tissue thermogenesis. In this review, we discuss recent advances in the role of miRNAs in adipocyte thermogenesis and obesity. The potential for miRNA-based therapies for obesity and recommendations for future research are highlighted, which may help provide new targets for treating obesity and obesity-related diseases.

## 1. Introduction

Obesity is an urgent public health issue worldwide. According to World Health Assembly, in May 2022 (https://www.worldobesity.org (accessed on 10 September 2022)), one billion people will be obese worldwide, including one in five women and one in seven males. When the body mass index (BMI) is 30 kg/m^2^ or higher, a person is considered obese. An increase in all-cause mortality is linked to a higher BMI, driven mainly by excess cardiovascular disease [1]. During obesity, proinflammatory M1 macrophages are increased in adipose tissue and associated with adipose tissue inflammation and insulin resistance [2]. By contrast, in lean humans and mice, relatively high numbers of anti-inflammatory M2 macrophages secrete anti-inflammatory cytokines and utilize oxidative metabolism to maintain adipose homeostasis [2]. Obesity is also associated with significant morbidity from multiple health problems, including hypertension, type 2 diabetes mellitus (T2D), cerebrovascular disease, kidney disease, and many types of cancers [3], as well as a variety of musculoskeletal illnesses [4]. As a result, obesity is highly associated with long-term physical impairments in populations worldwide. Given the health impact of obesity, it is vital to take action on obesity prevention and treatment. Traditional methods to treat obesity include changing dietary habits and increasing physical activity. However, such weight loss is often ineffective, as fat mass loss is often only temporary, and weight regain following long-term dieting and exercise is common [5]. Bariatric surgery is considered an effective alternative for obesity. Though, metabolic bone disease caused by bariatric surgery can lead to significant and ongoing bone loss [6,7].

Aiming to better understand the occurrence and treatment of obesity has brought adipose tissue to the center of metabolic disease discussion. Adipose tissue is a metabolically active organ that plays a key role in regulating whole-body energy homeostasis [8].

Based on tissue color and unique metabolic properties, adipose tissue has been divided into two primary categories: brown adipose tissue (BAT) and white adipose tissue (WAT). WAT is essential for energy storage. The production of non-shivering heat by BAT, which utilizes energy, is necessary for maintaining body temperature [9]. Since the adult human BAT was first identified [9,10,11], there has been growing interest in finding obesity treatments starting with the fat itself. Converting energy-storing WAT into energy-consuming brown fat may be an effective and harmless solution to obesity. Cold exposure and β-adrenergic agonist treatment activate classical BAT and promote the browning of WAT. Physiological stimuli for beige adipocyte formation include cold exposure, diet, and exercise [12]. Indeed, during the last decade, cold-induced human BAT activation and recruitment have demonstrated that non-shivering thermogenesis increases energy expenditure and ultimately helps reduce body fat [13,14,15]. These results point to the possibility of non-shivering thermogenesis, which involves the activation and recruitment of thermogenic adipocytes to boost energy expenditure in adipose organs [16]. Brown and beige thermogenic adipocytes have more mitochondria than white adipocytes, and uncoupling protein 1 (UCP1) is substantially abundant in these mitochondria [17]. UCP1 has the function of separating substrate oxidation from adenosine triphosphate (ATP) synthesis, which creates heat. After exposure to cold, beige adipocytes are recruited in the white adipose tissue, a process known as WAT “browning” [18]. Since UCP1-promoted thermogenesis consumes a lot of energy, thermogenic adipocytes are critical for energy balance and thus promote the loss of body weight [19]. Therefore, it has become more important to look for molecular mechanisms and internal cues that control the development of thermogenic adipocytes [20]. The transcriptional regulators PR domain containing 16 (PRDM16), Peroxisome proliferator-activated receptor γ2 (PPARγ2), and other elements have been identified as prospective targets at the moment [21]. PRDM16 function as maintains brown adipose tissue morphology and thermogenesis [22]. PPARγ is a transcription factor highly expressed in adipose tissue and considered a master regulator of adipogenesis in mammals. PPARγ activation upregulates adiponectin and downregulates some proinflammatory gene expression [23]. PPARγ expression is increased in subcutaneous adipose tissue (SAT) in obese subjects, whereas its expression is decreased in obese subjects with metabolic alterations like insulin resistance [24,25]. This suggests that changes in PPARγ expression may function as an adaptive mechanism in the development of obesity, and other metabolic changes may influence its expression.

MicroRNAs (miRNAs) are small, highly conserved non-coding RNA molecules that play a role in gene expression regulation. Additionally, miRNAs have been identified in exosomes, which can be ingested by nearby or distant cells and then regulate recipient cells [26]. Recently, miRNAs have been recognized as regulators of adipose function by directly or indirectly regulating adipose thermogenesis. Studies have shown that the up-or down-regulation of certain specific miRNAs is the key to affecting adipose thermogenesis. miRNAs also have important regulatory roles in various other biological processes, including regulating energy and lipid metabolism in diabetes and obesity [27]. MicroRNAs are brand-new regulators of the growth and operation of adipose tissue. As a result of numerous research illuminating the function of miRNAs in adipocyte formation and metabolism, these molecules have emerged as promising therapeutic targets to address the adverse effects of adipose tissue enlargement. This review discusses miRNAs involved in adipose tissue thermogenesis and proposes their novel therapeutic potential in the prevention and treatment of obesity.

## 2. MicroRNAs as Regulators of Adipose Thermogenesis

### 2.1. Positive Role of MicroRANs in Adipose Thermogenesis

#### 2.1.1. miR-30b/c

MicroRNA-30 (miR-30) family is a significant component of the microRNA family. There are five members of the miR-30 family: miR-30a, miR-30b, miR-30c, miR-30d, and miR-30e. MiR-30b/c, a member of the miRNA30 family, is strongly expressed in brown fat and can promote adipogenesis, according to recent studies [28,29]. The levels of MiR-30b/c are significantly raised during brown adipocyte development [29]. It has been demonstrated that exposure to cold or stimulation of adrenergic receptors causes the production of miR-30b and miR-30c [29]. In brown adipocytes and primary subcutaneous adipocytes, overexpression of miR-30b/c significantly raises the mRNA levels of UCP1 and cell death-inducing DFFA-like effector A (Cidea), two highly expressed thermogenic makers in BAT. By contrast, suppression of miR-30 downregulates UCP1 expression in vivo and in vitro [29]. Additionally, miR-30b/c targets the 3′-UTR of the receptor-interacting protein 140 (RIP140), a corepressor of thermogenic genes, and functions as a positive regulator of thermogenesis and the browning process of WAT [29,30]. The functions of the miR-30 family in the field of adipose thermogenesis and adipose metabolism need to be revealed in future studies to provide effective targets for the treatment of obesity.

#### 2.1.2. miR-32

MicroRNA-32 (miR-32) is an androgen receptor (AR)-regulated microRNA found on chromosome 9 in intron 14 of c9orf5, the gene encoding transmembrane protein 245 (TMEM245) [31]. In both mice and humans, miR-32 is expressed in many organs, including the brain, liver, kidney, serum, and breast tissues. miR-32 is crucial to the development of many malignancies as well as several illnesses [32]. The role of miR-32 in lipid metabolism was proposed as early as 2012 [33], but its role in fat thermogenesis has only been discovered in recent years. In mice, exposure to cold causes an upregulation of miR-32, a BAT-specific super-enhancer-associated miRNA. Ng et al. [34] revealed the function of miR-32 as a regulator of thermogenesis in brown fat. Their study found that inhibition of miR-32 in vivo by injection of miR-32 antisense oligonucleotides (ASO) resulted in impaired thermogenic response and decreased core body temperature after cold exposure. The BAT of cold-exposed animals given miR-32-ASO injections did not exhibit appreciable morphological changes compared to control mice, although UCP1 expression levels markedly decreased. Fibroblast growth factor 21 (FGF21) is an endocrine molecule normally produced by the liver with multiple metabolic effects, including enhanced browning of inguinal white adipose tissue (iWAT) [35]. By blocking the tumor suppressor Tob1 and activating the p38/MAPK signaling induced by cold exposure, miR-32 directly stimulates the BAT thermogenic program and FGF21 production both in vivo and in vitro [34].

#### 2.1.3. miR-455

MicroRNA 455 (miR-455) is an RNA gene mainly associated with Pettigrew syndrome and thyroid cancer. Following treatment with bone morphogenetic protein 7 (BMP7), which has been proven to increase brown fat mass and promote brown adipocyte differentiation, miR-455 was initially identified as a crucial regulator of brown adipogenesis in multipotent mesenchymal cells [36,37,38]. Following in vitro and in vivo experiments, miR-455 was identified as a downstream effector of BMP7 and a genuine BAT marker for humans and rodents [34]. Furthermore, adipose-specific gain-of-function miR-455 transgenic mice display increased browning of SAT upon cold exposure compared to controls [34]. The specific regulation mechanism is that miR-455 induces the expression of PPARγ by targeting and inhibiting Necdin and Runx1t1, respectively. C/EBPβ later activates PPARγ and PGC1α to recruit transcriptional complexes and other transcriptional regulators to elicit the expression of brown fat-specific genes [36]. There are few studies on miR-455 in adipose thermogenesis, and more research is needed to clarify its potential positive role in adipose thermogenesis in the future.

#### 2.1.4. miR-203

miR-203 was originally proposed for its ability to limit the stem cell potential of skin progenitor cells and to display tumor suppressor function in various cancers [39,40]. In 2014, Kim et al. [41] identified miR-203 as a novel regulator of brown adipocyte development. In primary brown adipocytes, inhibiting miR-203 reduced the expression of brown fat indicators (such as Ucp1, Pgc1, Cidea, and PPAR) and mitochondrial markers (such as Cox7 and Cox8) without changing the expression of common adipogenic markers, such as PPAR, Fabp4, and Adiponectin [41]. Next, new research further elucidates its important role in adipose thermogenesis. miR-203, according to Guo et al. [42], enhances glucose tolerance in high-fat diet (HFD) fed mice and encourages white adipose tissue browning in mice exposed to low temperatures. Mechanistically, miR-203 is triggered by cAMP-dependent C/EBP upregulation and suppresses IFN- signal pathway activation by directly targeting Lyn, a Jak1-Stat1 activator [42]. Additionally, their research offers mechanistic insights into the regulation of inflammatory response and sub-WAT browning by the cAMP-miR-203-IFN network, which may be used to develop novel treatments for obesity and metabolic syndrome [42].

#### 2.1.5. miR-182-5p

A miRNA that is expressed in adipose tissue and is significantly more abundant in differentiated brown adipocytes than in undifferentiated cells is called miR-182-5p [43]. Additional research revealed that in both mice and humans, adipose miR-182-5p levels were inversely associated with obesity [44]. More specifically, animals exposed to cold increased the expression of miR-182-5p in adipose tissue, whereas genes involved in mitochondrial biogenesis and thermogenesis were inhibited when miR-182-5p was deficient [44]. Mechanistically, the expression of Nr1d1 is suppressed by miR-182-5p, which then promotes the macrophage-dependent acetylcholine/PKA axis in adipocytes to encourage the browning of white adipose tissues [44]. These results demonstrate a pivotal role for miR-182-5p as a link between macrophages and adipocytes in C57BL/6 mice, which controls beige adipose thermogenesis.

#### 2.1.6. Other MicroRNAs as Positive Regulators of Adipose Thermogenesis

Several other microRNAs have important positive regulatory roles in adipose thermogenesis. For instance, the miRNA miR-337-3p is more abundant during brown adipocyte development and is strongly expressed in BAT relative to WAT [45]. The findings of Vonhögen et al. demonstrated that MiR-337-3p increased adipocyte thermogenesis by blocking Twist1, a substance that prevents brown adipocyte mitochondrial metabolism and activity [46]. The miR17-92 cluster is substantially abundant in BAT and stimulates beige cell production in SAT to maintain core body temperature in adipocyte-specific miR17-92 cluster overexpressed C57BL/6 mice model exposed to cold [47]. The functions of miR-124-3p in adipocyte thermogenesis were discovered by Li et al. [48]. They discovered that miR-124-3p overexpression increased thermogenesis by raising the number of mitochondria in brown adipocytes.

### 2.2. Negative Role of MicroRNAs in Adipose Thermogenesis

#### 2.2.1. miR-133

miR-133 was originally found to be expressed in BAT and SAT, markedly downregulated after cold exposure, with concomitant direct targeting and negative regulation of the key thermogenesis regulator PRDM16 [49,50,51]. Inhibiting miR-133 or its transcriptional regulator, myocyte enhancer factor-2 (Mef2), promoted brown adipocyte development and white adipocyte browning, which were associated with increased mitochondrial activity [50]. Furthermore, mice with a double deletion of miR-133a1 and miR-133a2 result in upregulated thermogenic gene programs in SAT [52]. A reduced level of miR-133a also improves body insulin sensitivity and glucose tolerance in vivo [52]. Reversine, a synthetic purine analog, promotes the expression of brown adipocyte marker genes and increases mitochondrial content. Kim et al. [53] recently reported that ectopic production of miR-133a reversed the browning effects of Reversine.

#### 2.2.2. miR-34a

MicroRNA 34a (miR-34a) has been identified as a tumor suppressor gene in various human malignancies. Its role in adipose has begun to gain attention in recent years. Previously published studies suggested the role of miR-34a in regulating the browning of WAT [54,55]. Increased miR-34a levels in obesity have been shown by Fu et al. [55] to limit the production of beige and brown fat. miR-34a knockdown in mice with diet-induced obesity by lentivirus enhanced the production of beige fat in all types of WATs and encouraged more browning in BAT [55]. miR-34a deficiency stimulates the production of the FGF21 receptor complex (FGFR1-KL) and SIRT1, which contributes to the activation of the browning transcriptional pathway via FGF21/SIRT1-dependent deacetylation of PGC-1 [55].

#### 2.2.3. miR-27

After cold exposure, miR-27 was identified as a key upstream regulator of the transcriptional network involved in beige and brown adipogenesis [56]. miR-27 expression is downregulated in BAT and iWAT, and the knockdown of miR-27 in iWAT increases the expression of thermogenic markers Ucp1, Prdm16, and Pgc1a [56]. Similarly, Kong et al. [57] demonstrated that miR-27b negatively regulates WAT browning by directly targeting Prdm16. Their study found that the expression of brown adipocyte markers (including Ucp1, Cidea, Cox8b, Cox7a1, and Prdm16) was significantly reduced in the experimental group by overexpressing miR-27b in stromal vascular fraction (SVF) extracted from SAT compared with the control group. In addition, Prdm16 and Ucp1 protein levels were also decreased in the miR-27b overexpressed group. These results imply that miR-27b may play a significant regulatory function in regulating adipose thermogenesis.

#### 2.2.4. miR-155

miR-155 is a multifunctional miRNA abundant in immune system cells and essential for immune responses [58]. Multiple studies identified C/EBPβ, an important pro-adipogenic transcription factor, as the target of miR-155 in many cells type, including white preadipocytes [59,60,61]. Chen et al. [62] clarified its role in regulating brown and beige adipocyte differentiation. In their study, the gain and loss function of miR-155 experiments indicated that miR-155 negatively regulates adipose browning [62]. In white adipocytes, miR-155 deficiency stimulates the thermogenic program. As a result, mice lacking miR-155 have a greater capacity to adjust to cold exposure and attract inducible brown/beige cells [62]. Interestingly, clinical studies have linked the expression of miR-155 to adipose tissue dysfunction and obesity, suggesting its potential role in humans [63]. Taken together, therapeutic attempts to reduce miR-155 may be a promising approach for treating obesity in humans.

#### 2.2.5. miR-327

Genome-wide miRNA profiling identified that miR-327 was significantly downregulated in stromal-vascular fraction (SVF) isolated from WAT under cold or β3-adrenergic stimulation [64]. In addition, miR-327 can directly bind to the 3′-untranslated region (UTR) of Fgf10 [64]. On the one hand, inhibition of miR-327 causes browning and boosts systemic metabolic rate in mice under thermoneutral conditions [64]. On the other hand, local miR-327 delivery to WAT by adenovirus dramatically reduces UCP1 expression and blocks cold-induced browning [64]. Collectively, this study of the role of miR-327 in adipose thermogenesis demonstrates that microRNAs can be used to treat obesity, T2DM, and other metabolic diseases. Future miRNAs targeting Fgf10 will likely provide an effective novel approach to obesity treatment.

#### 2.2.6. miR-494-3p

It has also been observed that miR-494-3p controls white adipose thermogenesis both in vivo and in vitro. miR-494-3p levels were dramatically downregulated in the iWAT following cold exposure, as shown by Lemecha et al. [65]. Further gain and loss of function studies of miR-494-3p in 3T3-L1 adipocytes showed that overexpressed miR-494-3p inhibits adipocyte browning, mitochondrial biogenesis, and thermogenesis through PGC1-α [65]. There are few studies on the role of miR-494-3p in adipose function, and more research is needed to further clarify the specific role of miR-494-3p in adipose thermogenesis.

#### 2.2.7. miR-199a/214 Cluster

To screen for miRNAs important for brown adipocyte differentiation, He and colleagues found that the expression of miR-199a and miR-214 was significantly reduced during brown adipocyte differentiation by miRNA microarray analysis [43]. miR-199a/214 cluster is a negative regulator of thermogenesis by directly targeting adipose browning transcriptional regulator PRDM16 and peroxisome PGC-1α [43]. According to studies, brown adipocyte differentiation is inhibited by overexpression of the miR-199a/214 cluster, suppressing the expression of thermogenic genes and mitochondrial respiration. In contrast, beige adipocytes with this cluster knocked down have higher levels of thermogenic gene expression and mitochondrial function [43].

#### 2.2.8. Other MicroRNAs as Negative Regulators of Adipose Thermogenesis

**Figure 1:**
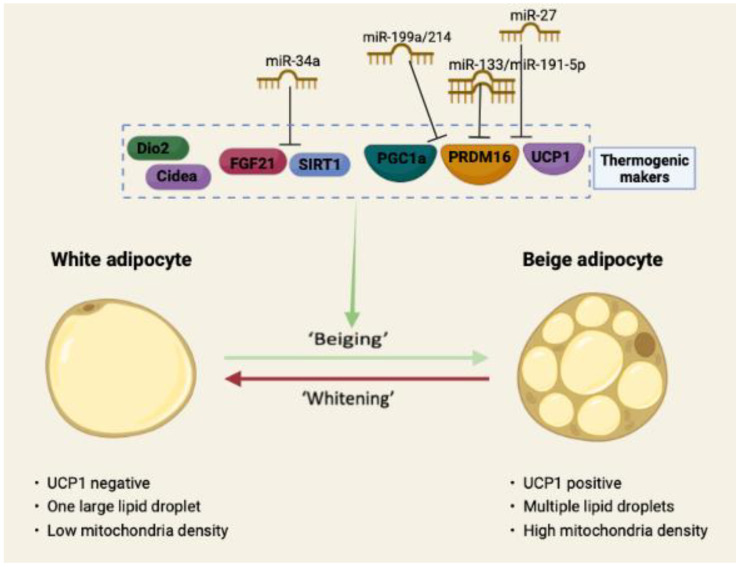
miRNAs inhibited adipocytes browning process. Regulation of the WAT beige process involves the action of a few specific proteins and nucleic acids. This trans-acting factor orchestra regulates genes related to mitochondrial biogenesis, cellular oxidative capacity, and non-shivering thermogenesis. The action of some microRNAs (such as miR-133, miR-34a, miR-27, etc.) inhibits the process of WAT browning.

Many other microRNAs have important negative regulatory roles in adipose thermogenesis. For example, Ding et al. [66] found that mice deficient in miR-149-3p improved inguinal fat thermogenesis and elevated total body energy expenditure. Additionally, it was shown that miR-23b-5p functions as a negative regulator in the regulation of the brown adipocyte thermogenic gene program [67]. It is interesting to note that a recent study revealed lowly expressed miR-191-5p in long-term exercise-secreted extracellular vesicles enhances the browning of WAT by adversely targeting PRDM16-3′UTR, hence increasing thermogenesis and lowering obesity [68]. The function of miR-191-5p in thermogenesis also needs to be examined further in models of cold or β-adrenergic receptor stimuli. Finally, the negative regulatory role of microRNAs in adipose thermogenesis can also be more intuitively understood through the schematic diagram in Figure 1.

### 2.3. MicroRNAs with Controversial Functions in Adipose Thermogenesis

#### 2.3.1. miR-22

The role of miR-22 in adipocytes was proposed by Huang et al. in 2012 [69]. Their research revealed miR-22 is a crucial regulator of the equilibrium between the adipogenic and osteogenic development of mesenchymal stem cells generated from human adipose tissue [69]. The function of miR-22 in adipocyte thermogenesis is controversial. According to Diniz et al. [70], the knockout of miR-22 in C57BL/6 mice by targeting exon2 exhibits increased energy expenditure and reduced fat mass accumulation brought on by HFD compared to WT mice. Another publication from the same group using the same miR-22 KO mice model, according to Lima et al. [71], led to WAT browning and protected mice from HFD-induced mitochondrial dysfunction in WAT and BAT. In addition, compared to wild-type mice, miR-22 KO mice fed with HFD displayed increased thermogenic gene expression and adrenergic signaling in BAT [71]. However, another study by Lou et al. [72] on the thermogenic function of miR-22 showed that both global and adipocyte-specific miR-22 deficiency in mice by targeting the second exon resulted in impaired cold adaptation and suppressed thermogenic genes expression in BAT by jointly stimulating the mTORC1 and glycolytic signaling pathways. More research is needed to elucidate the exact role of miRNA22 in adipose thermogenesis.

#### 2.3.2. miR-33

The hypothesis that miR-33 might regulate PGC1α-related brown adipocyte metabolism was raised after the gene encoding PGC1α, Ppargc1a, was discovered to be a miR-33 target gene in macrophages [73]. Subsequently, miR-33 was characterized by Afonso et al. [74] as a negative regulator of adaptive thermogenesis and white adipose tissue beiging. The level of miR-33 was reduced following cold exposure, and inhibiting miR-33 with miR-33 antisense oligonucleotides enhanced beige in white adipose tissue of C57BL6/J mice [74]. However, Horie et al. [75] showed that knockout of miR-33 displays impaired thermogenesis via reduced BAT activation in C57BL/6 mice. More data will be needed to further clarify the function of miR-33 in adipose thermogenesis and firmly identify how it affects adipose function.

## 3. MicroRNAs as Therapeutic Targets for Obesity

### 3.1. MicroRNAs Associated with Obesity

Obesity is a risk factor for insulin resistance and type 2 diabetes [76]. Type 2 Diabetes is inherently characterized by insulin resistance [76]. Insulin resistance (IR) is significantly influenced by elevated amounts of non-esterified fatty acids, glycerol, hormones, and proinflammatory cytokines produced in the adipose tissue of obese individuals [76]. Numerous clinical studies have discovered differences in miRNA expression in the adipose tissue of lean and obese individuals, suggesting that obesity may lead to altered miRNA expression and further metabolic changes [77,78,79,80,81,82,83]. For instance, Meerson et al. [79] found that BMI was positively associated with miR-221 level (especially in women) and negatively associated with miR-193a-3p and miR-193b-5p levels. The differences in these microRNAs in individuals with different BMI further reveal the potential role of microRNAs in the development of obesity. In addition, the study also found that miR-378, which is overexpressed in SAT of obese individuals and mature adipocytes, can directly target adiponectin [83,84]. In contrast, another microRNA called miR-126 was reported to be repressed in SAT in obese individuals by targeting C-C Motif Chemokine Ligand 2 (CCL2) [85]. CCL2 is a chemokine secreted by adipose tissue that activates IR-related inflammatory responses [86] and is regulated by miRNAs as well [87]. It has been demonstrated that miR-130a and miR-130b, members of the miR-130 family, are reduced in the SAT of obese people and directly target PPARγ [83,88]. Likewise, another microRNA called miR-519 was reported to be overexpressed in SAT in morbidly obese individuals by directly targeting PPARγ [82].

Obesity is widely recognized to cause an increase in free fatty acids (FFA) from diet or adipose tissue lipolysis. FFA binds to TLRs such as TLR4 and TLR2, further triggering the production of numerous proinflammatory chemokines, which stimulate downstream NF-κB activation [86,89]. Individuals with obesity have higher levels of TLR2 and TLR4 expression in their adipose tissue, indicating that these receptors are implicated in the inflammation-related signaling associated with obesity [90]. Visceral adipose microRNA-223 is elevated in both human and mouse obesity, according to research by Deiuliis et al. [77]. The results of their experiments specifically demonstrated that the stromal vascular cells of human VAT were responsible for increasing miRNA-233 expression. By regulating TLR4 expression, miR-223 significantly impacts macrophage penetration into adipose tissue [77]. miR-1934 inhibits the expression of IL6, IL1b, and CCL2, and its expression is suppressed in the VAT of obese individuals [78]. Serum resistin levels are elevated in obese subjects, and their concentrations are directly related to IR [91]. Resistin was found to be another target of miR-1934 [78].

**Figure 2:**
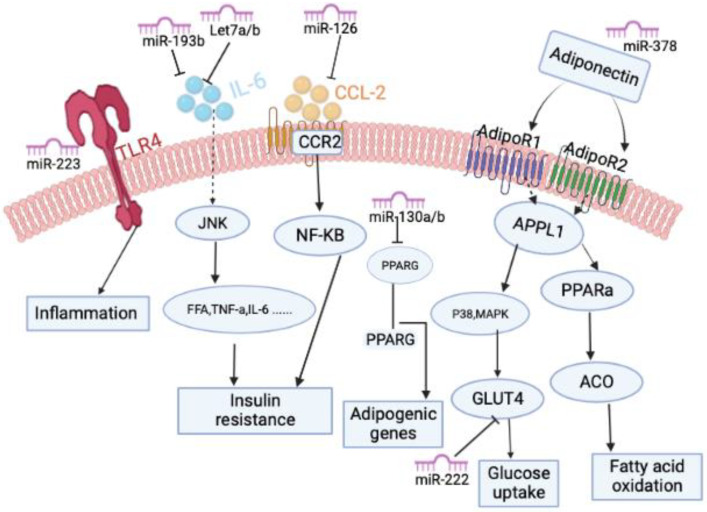
Molecular pathway of miRNAs as therapeutic targets for obesity. MicroRNAs can affect obesity-related signaling pathways by regulating the activation of transcription factors and cellular proteins.

Several studies have found that reduced plasma levels of several members of the let-7 miRNA family are associated with obesity and T2DM [92,93]. Moreover, studies have shown that the expression of let-7a and let-7d is reduced in the SAT of obese individuals compared to lean individuals [80]. Interestingly, another study discovered that the microRNA let-7 directly targets the gene IL-6 and that overexpressing let-7 by transfecting let-7 precursors decreased IL-6 expression [94]. Taken together, obesity-induced inflammation may be mediated by reduced expression of the let-7 family of miRNAs. Similarly, another microRNA called miR-193b was also found to have reduced expression in obesity [79,80]. Obesity is associated with decreased adiponectin expression in plasma and adipose tissue, and adiponectin reduces skeletal muscle IR by promoting FFA oxidation [95,96,97]. Belarbi et al. [87] discovered that miR-193b expression in SAT correlated with adiponectin gene expression and insulin resistance. It is known that Interleukin 6 (IL-6) induces IR in adipose tissue [98]. It was also found that miR-193b overexpression in human subcutaneous adipocytes attenuated IL-6 secretion [80]. miR-193b regulated CCL2 production through a transcription factors network (MAX, ARNT, BHLH, and ETS1); many of those transcription factors have been identified in other inflammatory conditions [80]. Collectively, these results suggest that obesity-associated reduction in miR-193b expression leads to the development of IR by reducing adiponectin expression and increasing IL6 and CCL2 expression. The detailed mechanism diagram can also be seen in Figure 2.

Studies have revealed a connection between obesity and the expression of two miR-221 family members, miR-221 and miR-222 [79,80,83]. According to a recent clinical investigation by Ojeda-Rodrguez et al. [83,99], obese people showed higher miR-221 expression in SAT than people of normal weight did. Obese subjects who lost significant weight after surgery had reduced miR-221 expression in SAT [100]. Surprisingly, miR-221 circulating levels were considerably lower in obese patients compared to normal-weight subjects. The study found that miR-221 was reduced in the plasma of obese children compared with normal-weight children and that weight loss resulted in increased plasma miR-221 levels [101]. Additionally, plasma miR-221 was reduced in morbidly obese subjects [102,103]. Conversely, another study found elevated serum miR-221 levels in women with metabolic syndrome compared with controls [104]. Based on this, future research needs to focus more on explaining the differences between miRNAs in adipose tissue and circulating levels of obese subjects. It has been established that obesity and T2DM are associated with reduced GLUT4 expression in SAT [105]. Shi et al. [106] demonstrated that overexpressed miR-222 in 3T3-L1 adipocytes decreased GLUT4 expression. On the one hand, studies have also demonstrated that inflammation plays a role in regulating the expression of miR-221 [100,107]. On the other hand, miR-221 is also closely related to the IR process regulated by adiponectin [79,108]. Overall, miR-221 is a particularly promising target in obesity treatment, which requires more research to reveal its specific potential application mechanism.

### 3.2. Adipokines Regulate miRNA Activity in Obesity

Adipokines are cell signaling molecules produced by adipose tissue, and they play important roles in regulating body metabolisms such as energy metabolism, inflammation, and obesity. Adipokines mainly include leptin, adiponectin, resistin, interleukin-6, and many others. Numerous studies have shown a non-negligible interaction between microRNAs and adipokines to jointly maintain metabolic homeostasis.

Several in vivo and in vitro studies have shown that leptin regulates miRNA expression in various human tissues. Animal studies have shown altered expression of many microRNAs in leptin-deficient ob/ob mice or T2D db/db mice, such as miR-184 and miR-103 [109,110]. A clinical study also showed that in obese patients, the expression of miR-27b and miR-145 was positively correlated with the expression and activity of the leptin receptor [111]. In addition, many studies have also found that adiponectin and resistin are also involved in regulating the expression of many microRNAs in obesity-related conditions [112,113,114,115].

Many studies have found that the roles of inflammatory adipokines such as TNFα, IL6, and IL1β in obesity-related diseases are attributed to altered miRNA profiles. For example, Shi et al. [116] found that TNFα and IL6 induce an inflammatory response to obesity in adipose tissue by upregulating miR-146b. Similarly, some clinical studies have also confirmed the regulation of inflammatory adipokines on microRNAs. Nteeba et al. [117] found that miR-125b and miR143 levels were negatively correlated with IL1β, IL6, and TNFα mRNA levels in the ovaries of obese individuals. Moreover, increased miR-146a-5p expression was associated with elevated levels of inflammatory adipokines such as TLR4, NFκB, IL6, and TNFα in obese patients [118].

### 3.3. Influence of Obesity in the miRNome

Many omics analyses have well-revealed microRNAs associated with obesity. Herrero-Aguayo et al. [119] found that a total of 26 microRNAs were altered in the plasma of obese subjects (*n* = 4/body mass index > 30) compared to controls (*n* = 4/body mass index < 25) using the Affymetrix-miRNA array (*p* < 0.01). Among these microRNAs, the most striking difference was miR-4454, which levels are consistently higher in obese patients and are associated with insulin resistance [119]. Additionally, Kuryłowicz et al. [120] applied next-generation sequencing (NGS) to identify visceral (VAT) and SAT samples from 47 normal-weight, obese, and obese after surgery-induced weight loss individuals with differentially expressed microRNAs (miRNAs). The results identify molecular pathways differentially regulated in the adipose tissue of normal-weight and obese subjects before and after weight loss [120]. Another omics analysis found dysregulated expression of multiple miRNAs, such as miR-200a/200b, in epididymal WAT of ob/ob mice compared to WT mice [121]. These miRNome-based analyses provide us with more obesity-related microRNA targets, which provide directions for more experimental studies on microRNA-based obesity treatment. In addition, correlative omics analysis based on measuring changes in miRNA levels during weight loss (after bariatric surgery or lifestyle intervention) is also required in the future to screen for microRNAs that can be targeted for obesity therapy.

### 3.4. Novel Approaches of MicroRNAs Delivery

Since miRNAs are involved in regulating many key factors in the development of obesity, the use of miRNAs for obesity treatment is receiving extensive attention. Regarding the delivery system of microRNAs, there are generally two main categories, viral-based miRNA delivery system, and non-viral-based miRNA delivery system.

Transgenic viruses can deliver desired oligonucleotides and anti-miRs to increase or suppress the expression of target miRNAs [122]. Viruses used in virus-based miRNA delivery systems include lentiviruses, retroviruses, adenoviruses, and adeno-associated viruses (AAV). Currently, virus-mediated miRNA delivery has proven promising in treating many diseases. For example, Kasar et al. [123] found systemic in vivo lentiviral delivery of miR-15a/16 reduced malignancy in a mouse model of chronic lymphocytic leukemia. In addition, another study used an AAV delivery system with high transduction efficiency to overexpress miR-298 and alleviate neuromuscular disease in a mouse model [124]. These promising results illustrate the promise of using viral-based delivery systems to deliver miRNAs to treat obesity.

Although viral-based miRNA delivery systems are highly efficient, they are associated with high immunogenicity, toxicity, and size limitations. The less toxic and biocompatible non-viral-based miRNA delivery system ensures successful intracellular delivery of miRNA or miRNA expression vectors without degradation by nucleases. Non-viral-based miRNA delivery methods mainly include lipid, polymer, inorganic, and extracellular vesicle-based methods. Lipid-based nanocarriers are currently the most widely used non-viral delivery method [125]. Multiple studies have demonstrated the successful transport of lipid complexes in vivo to treat various diseases [126,127,128].

With the popularity of miRNA therapy in treating various human diseases, more and more new oligonucleotide delivery strategies have emerged to improve the therapeutic effect. Argininocalix [4] arene, a novel synthetic cationic surfactant, can efficiently transfer miRNA and anti-miRNA molecules to target cells in vitro [129,130]. A compound called Neuromag^®^ has also been shown to have a good effect on microRNA delivery [131,132]. In addition, the researchers designed a nanobody-functionalized nucleic acid nanogel for the targeted delivery of miRNAs to cells to treat tumors [133,134,135]. Recently, new studies have found that the use of outer membrane vesicles (OMVs) of *Escherichia coli* as a delivery system for miRNA transport in nanoscale spherical vesicles is promising in cancer therapy [136]. Moreover, using exosomes as miRNA delivery vehicles may effectively overcome miRNA degradation in vivo, which has also been shown to promote cancer cell apoptosis [137]. Collectively, these new microRNA delivery methods provide a new direction for further application in microRNA-targeted obesity treatment.

## 4. Summary and Perspective

In this review, we highlight the diverse roles of microRNAs in adipose thermogenesis and identify their regulatory roles in the development of obesity (also refer to Table 1). In the future, we anticipate that microRNAs will be exploited to treat obesity.

Over the past decades, BAT has gained increasing attention as a potential target for novel therapeutic tools to combat obesity [138]. Inducible brown adipocytes (known as beige or brite adipocytes) in the WAT depot have also received more attention in recent years due to higher energy expenditure capabilities and positive effects on preventing diet-induced obesity and T2D [139]. Like other hormones or molecules, miRNAs play an important role in brown adipogenesis [140]. Significant progress has been made in understanding the physiological activities of miRNAs in adipocytes, as well as their pathological implications in obesity and accompanying metabolic problems. In vitro and in vivo studies have shown that miRNAs can positively or negatively affect brown/beige adipogenesis. At present, the role of miRNAs in adipose thermogenesis is mainly to study its regulation on some thermogenic genes, including some marker thermogenic genes like UCP1. UCP1 is undoubtedly a significant driver of thermogenesis. Nonetheless, many studies have identified several UCP1-independent thermogenic pathways shown to be involved in the regulation of whole-body energy homeostasis [8]. For example, Ca^2+^ cycling thermogenesis and creatine cycling-related thermogenesis [141,142]. Then, in future research on microRNAs related to thermogenesis, it is also necessary to study their effects on UCP1-independent thermogenic pathways, which will enable us to have a more comprehensive and in-depth understanding of how microRNAs can be used in the targeted treatment of obesity.

Although miRNA-targeted medicines have paved the way for treating different diseases, they still face a formidable obstacle in their development. Furthermore, the application of miRNA treatment in vivo in adipose tissue is currently understudied since adipocytes are barely responsive to exogenous nucleotide transfer due to a significant number of intracellular lipid droplets [143]. Future studies may try to induce or reduce miRNA expression using viral or liposome tissue-specific delivery to further apply it to clinical therapy. Furthermore, direct microinjecting miRNAs into adipocytes may be another way to avoid these obstacles. Despite these challenges, the development of studies related to the omics identification and characterization of miRNAs associated with adipogenesis and obesity also provides new therapeutic targets.

On the other hand, because miRNAs may be carried in body fluids such as blood, they are anticipated to play an essential role in metabolic interaction between different cell types inside adipose tissue [144]. Additionally, adipose tissue is a significant source of circulating exosomal miRNAs, indicating that miRNAs are crucial for controlling gene expression in other connected tissues [144,145]. It shows that circulating miRNAs could be valuable biomarkers of brown/beige fat activity and potential treatment approaches for obesity.

Taken together, studies to date on miRNAs in adipose development suggest that while the development of miRNA-based therapies is still in its infancy, miRNAs still have potential in the field of obesity-related diseases due to well-characterized miRNA overexpression and inhibition methods, and the potency of miRNAs as genetic regulators. The presence of circulating miRNAs in patient blood creates the possibility that miRNAs may serve as exciting novel diagnostic markers related to adipogenesis and obesity. In the case of metabolic syndrome, it may also be used in conjunction with other treatments, such as anti-inflammatory therapy, to reduce obesity and its attendant complications. Despite the growing understanding of miRNA functions in adipose, further studies are needed to fully understand the complex mechanisms behind miRNA-mediated regulation of adipose thermogenesis for developing therapeutic approaches for obesity.

## Figures and Tables

**Table 1:** Summary of miRNAs involved in adipose thermogenesis.

microRNA	Cell/Tissue	Effect of Thermogenesis	Reference(s)
miR-30b/c	WAT/primary adipocytes	Positive	[29,30]
miR-32	BAT	Positive	[34]
miR-203	WAT/brown adipocytes	Positive	[41,42]
miR-337-3p	BAT/brown adipocytes	Positive	[45]
miR17-92	BAT	Positive	[47]
miR-124-3p	Brown adipocytes	Positive	[48]
miR-182-5p	WAT/primary adipocytes	Positive	[43,44]
miR-455	WAT/primary adipocytes	Positive	[36]
miR-133	BAT/WAT	Negative	[50,52,53]
miR-327	WAT	Negative	[64]
miR-149-3p	WAT	Negative	[66]
miR-199a/214	Primary adipocytes	Negative	[43]
miR-494-3p	WAT/primary adipocytes	Negative	[65]
miR-191-5p	WAT	Negative	[68]
miR-27	BAT/WAT	Negative	[56,57]
miR-155	WAT/primary adipocytes	Negative	[59,60,61,62]
miR-34a	BAT/WAT	Negative	[55]
miR-23b-5p	Brown adipocytes	Negative	[67]
miR-22	BAT/WAT/adipocytes	Controversial	[70,71,72]
miR-33	BAT/WAT	Controversial	[74,75]

## Data Availability

Not applicable.
